# Cancer Incidence among Glyphosate-Exposed Pesticide Applicators in the Agricultural Health Study

**DOI:** 10.1289/ehp.7340

**Published:** 2004-11-04

**Authors:** Anneclaire J. De Roos, Aaron Blair, Jennifer A. Rusiecki, Jane A. Hoppin, Megan Svec, Mustafa Dosemeci, Dale P. Sandler, Michael C. Alavanja

**Affiliations:** ^1^Program in Epidemiology, Fred Hutchinson Cancer Research Center and the Department of Epidemiology, University of Washington, Seattle, Washington, USA; ^2^Division of Cancer Epidemiology and Genetics, National Cancer Institute, National Institutes of Health, Department of Health and Human Services, Bethesda, Maryland, USA; ^3^Epidemiology Program, National Institute of Environmental Health Sciences, National Institutes of Health, Department of Health and Human Services, Research Triangle Park, North Carolina, USA

**Keywords:** cancer, cohort study, farming, glyphosate, pesticide

## Abstract

Glyphosate is a broad-spectrum herbicide that is one of the most frequently applied pesticides in the world. Although there has been little consistent evidence of genotoxicity or carcinogenicity from *in vitro* and animal studies, a few epidemiologic reports have indicated potential health effects of glyphosate. We evaluated associations between glyphosate exposure and cancer incidence in the Agricultural Health Study (AHS), a prospective cohort study of 57,311 licensed pesticide applicators in Iowa and North Carolina. Detailed information on pesticide use and other factors was obtained from a self-administered questionnaire completed at time of enrollment (1993–1997). Among private and commercial applicators, 75.5% reported having ever used glyphosate, of which > 97% were men. In this analysis, glyphosate exposure was defined as *a*) ever personally mixed or applied products containing glyphosate; *b*) cumulative lifetime days of use, or “cumulative exposure days” (years of use × days/year); and *c*) intensity-weighted cumulative exposure days (years of use × days/year × estimated intensity level). Poisson regression was used to estimate exposure–response relations between glyphosate and incidence of all cancers combined and 12 relatively common cancer subtypes. Glyphosate exposure was not associated with cancer incidence overall or with most of the cancer subtypes we studied. There was a suggested association with multiple myeloma incidence that should be followed up as more cases occur in the AHS. Given the widespread use of glyphosate, future analyses of the AHS will allow further examination of long-term health effects, including less common cancers.

Glyphosate [*N*-(phosphonomethyl)glycine], commonly sold in the commercial formulation named Roundup (Monsanto Company, St. Louis, MO), has been a frequently used herbicide on both cropland and noncropland areas of the world since its introduction in the 1970s (Williams et al. 2000). Roundup is a combination of the active ingredient and other chemicals, including a surfactant (poly-oxyethyleneamine) that enhances the spreading of spray droplets when they contact foliage. Glyphosate is a broad-spectrum herbicide of which the primary mechanism is inhibition of the enzyme 5-enolpyruvoyl-shikimate 3-phosphate synthase, which is essential for the formation of aromatic amino acids in plants (Steinrucken and Amrhein 1980). Because this specific biologic pathway operates only in plants and microorganisms, the mechanism is not considered to be a risk for humans. Nevertheless, genotoxic, hormonal, and enzymatic effects in mammals have been reported (Bolognesi et al. 1997; Daruich et al. 2001; El Demerdash et al. 2001; Hietanen et al. 1983; Lioi et al. 1998a, 1998b; Olorunsogo et al. 1979; Peluso et al. 1998; Walsh et al. 2000; Yousef et al. 1995).

Results from genotoxicity studies of glyphosate have been conflicting. Glyphosate did not show any genotoxic activity in a battery of assays (Garry et al. 1999; Grisolia 2002; Li and Long 1988; Wildeman and Nazar 1982). However, other studies observed that glyphosate treatment of human lymphocytes *in vitro* resulted in increased sister chromatid exchanges (Bolognesi et al. 1997), chromosomal aberrations (Lioi et al. 1998b), and indicators of oxidative stress (Lioi et al. 1998b). Some studies found slightly greater toxicity of the Roundup formulation compared with glyphosate, in terms of both acute toxicity (Folmar et al. 1979; Martinez et al. 1990; Mitchell et al. 1987) and genotoxicity (Bolognesi et al. 1997; Vigfusson and Vyse 1980). Roundup was associated with increased DNA adducts in mice (Peluso et al. 1998) and a weak mutagenic effect in the *Salmonella* assay (Kale et al. 1995; Moriya et al. 1983; Rank et al. 1993), whereas glyphosate alone did not show these effects. Chronic feeding studies of glyphosate have not provided evidence of a carcinogenic effect in mice or rats (Williams et al. 2000).

The U.S. Environmental Protection Agency (U.S. EPA 1993) and the World Health Organization (WHO 1994) reviewed the toxicology data on glyphosate and concluded that glyphosate is not mutagenic or carcinogenic. The U.S. EPA classified glyphosate as category E, indicating “evidence of noncarcinogenicity for humans” (U.S. EPA 1993). Despite this conclusion, three recent case–control studies suggested an association between reported glyphosate use and the risk of non-Hodgkin lymphoma (NHL) (De Roos et al. 2003b; Hardell and Eriksson 1999; Hardell et al. 2002; McDuffie et al. 2001). Considering the widespread and frequent use of glyphosate in both the United States and the rest of the world, ongoing risk assessment is of importance. We studied site-specific cancer incidence associated with glyphosate use among pesticide applicators in the Agricultural Health Study (AHS) cohort.

## Materials and Methods

### Cohort enrollment and follow-up.

The AHS is a prospective cohort study in Iowa and North Carolina, which includes 57,311 private and commercial applicators who were licensed to apply restricted-use pesticides at the time of enrollment. Recruitment of the applicators occurred between 1993 and 1997 (Alavanja et al. 1996). Cohort members were matched to cancer registry files in Iowa and North Carolina for case identification and to the state death registries and the National Death Index (National Center for Health Statistics 1999) to ascertain vital status. Incident cancers were identified for the time period from the date of enrollment until 31 December 2001 and were coded according to the *International Classification of Diseases,* 9th Revision (WHO 1977). If cohort members had moved from the state, they were censored in the year they left. The median time of follow-up was 6.7 years.

### Exposure assessment.

Using a self-administered enrollment questionnaire, we collected comprehensive-use data on 22 pesticides, ever/never use information for 28 additional pesticides, and general information on pesticide application methods, personal protective equipment, pesticide mixing, and equipment repair. Data were also collected on basic demographic and lifestyle factors. Applicators who completed this questionnaire were given a self-administered take-home questionnaire, which contained additional questions on occupational exposures and lifestyle factors. The questionnaires are available from the AHS website (National Institutes of Health 2004).

We constructed three glyphosate exposure metrics for this analysis: *a*) ever personally mixed or applied products containing glyphosate (ever/never); *b*) cumulative lifetime days of use, or “cumulative exposure days” (years of use × days per year, categorized in tertiles among users: 1–20, 21–56, 57–2,678); and *c*) intensity-weighted cumulative exposure days (years of use × days per year × intensity level, categorized in tertiles: 0.1–79.5, 79.6–337.1, 337.2–18,241). Tertiles were chosen *a priori* as the cut points with which to categorize exposure data, to avoid sparse data for rare cancers in the high-exposure categories. Intensity levels were estimated using questionnaire data from enrollment and measurement data from the published pesticide exposure literature, as follows: intensity level = [(mixing status + application method + equipment repair status) × personal protective equipment use] (Dosemeci et al. 2002).

### Data analysis.

Persons whose first primary cancer occurred before the time of enrollment (*n* = 1,074) were excluded from analyses, as were subjects who were lost to follow-up or otherwise did not contribute any person-time (*n* = 298) and applicators who did not provide any information on age (*n* = 7) or whether they had ever used glyphosate (*n* = 1,678). After exclusions, 54,315 subjects were available for inclusion in the age-adjusted analyses of cancer incidence in relation to glyphosate use; however, other analyses contained fewer observations because of missing data for duration and frequency of glyphosate use or for covariates.

We compared certain baseline characteristics among three types of pesticide applicators: *a*) those applicators who never personally used glyphosate; *b*) applicators with the lowest glyphosate exposure, defined as being in the lowest tertile of cumulative exposure days; and *c*) those with higher glyphosate exposure, defined as being in the middle or highest tertile of cumulative exposure days. The purpose of the comparison was to identify potential confounders of glyphosate exposure–disease associations for the various analyses we conducted. Differences between the exposure groups were tested using the chi-square statistics and associated *p*-values.

Poisson regression analyses were carried out for all cancers combined and specific cancer sites to estimate rate ratios (RRs) and 95% confidence intervals (CIs) associated with glyphosate exposure metrics; the effect of each metric was evaluated in a separate model for each cancer. We analyzed tertile exposure variables in separate models using either the lowest-tertile–exposed or never-exposed subjects as the reference category. We investigated specific cancer sites for which there were at least 30 cases with sufficient information for inclusion in age-adjusted analyses. These cancers were then evaluated for all the exposure metrics and in adjusted analyses, despite smaller numbers of cases upon further adjustment. For each exposure metric, RRs were adjusted for demographic and lifestyle factors, including age at enrollment (continuous), education (dichotomous: ≤high school graduate or GED/education beyond high school), pack-years of cigarette smoking [indicator variables: never, pack-years at or below the median (12 pack-years), pack-years above the median], alcohol consumption in the past year [indicator variables: none, frequency at or below the median (72 drinks), frequency above the median], family history of cancer in first-degree relatives (dichotomous: yes/no), and state of residence (dichotomous: Iowa/North Carolina). There was insufficient variability in sex or applicator type to adjust for these factors.

Potential confounding from exposure to other pesticides was explored by adjusting for the five pesticides for which cumulative-exposure-day variables were most highly associated with glyphosate cumulative exposure days [(2,4-dichlorophenoxy)acetic acid (2,4-D), alachlor, atrazine, metolachlor, trifluralin]; these pesticide exposures were coded as variables indicating never, low, and high, with the split between low and high as the median of their cumulative exposure days. Additionally, of the pesticides for which only ever/never use information was available, we adjusted for the five pesticides that were most highly associated with ever use of glyphosate (benomyl, maneb, paraquat, carbaryl, diazinon). Where inclusion of all 10 other pesticides in a model changed a glyphosate exposure estimate by at least 20% (compared with a model restricted to the same observations), these results were presented as the final results for that cancer; otherwise, estimates adjusted only for demographic and lifestyle factors are presented.

Tests for trend across tertiles were conducted by creating a continuous variable with assigned values equal to the median value of cumulative exposure days (or intensity-weighted exposure days) within each tertile; the *p*-value for the trend test was that from the Poisson model coefficient for this continuous variable. We considered *p*-values < 0.10 as indicative of a trend.

Additional analyses were conducted for cancers for which we observed elevated RRs, and for NHL because of its association with glyphosate in previous studies. These included analyses stratified by state and analyses across quartiles and quintiles (where numbers allowed) of exposure days metrics.

## Results

Selected characteristics of the glyphosate-exposed and never-exposed applicators are presented in Table 1. Among 54,315 subjects included in age-adjusted analyses, 41,035 (75.5%) reported having ever personally mixed or applied products containing glyphosate, and 13,280 (24.5%) did not. The cohort, both exposed and never exposed, was composed of primarily of male, middle-aged, private applicators. This is a population with relatively low smoking prevalence; in both the exposed and never-exposed groups, more than half of the subjects reported that they had never smoked. Significant differences (*p* < 0.05) existed between never-exposed and lowest-exposed subjects for all of the characteristics in Table 1. Lowest- and higher-exposed subjects (*p* < 0.05) also differed on several factors, the most notable being that higher-exposed subjects were more likely to be commercial applicators, to have consumed greater amounts of alcohol in the past year, and to have used other specific pesticides. However, lowest- and higher-exposed subjects were similar to each other (*p* ≥0.05) in characteristics including smoking and family history of cancer in a first-degree relative. In addition, lowest- and higher-exposed subjects were more similar to each other than to their never-exposed counterparts (by qualitative comparison of percentages only) in factors including North Carolina residence, education beyond high school, and use of other pesticides. Because of relative similarities between lowest- and higher-exposed in factors associated with socioeconomic status and other exposures, we decided to conduct some analyses using lowest-exposed rather than never-exposed applicators as the reference group, in order to avoid residual confounding by unmeasured covariates. However, we decided *a priori* that any association should be apparent regardless of which reference group was used.

RRs for the association of all cancers combined and specific cancers with having ever used glyphosate are presented in Table 2. RRs adjusted for age only are presented, as well as RRs adjusted for demographic and lifestyle factors and, in some cases, for other pesticides. The incidence of all cancers combined was not associated with glyphosate use, nor were most specific cancers. There was an 80% increased risk of melanoma associated with glyphosate use in the age-adjusted analysis, which diminished slightly upon further adjustment. Adjusted risk estimates for colon, rectum, kidney, and bladder cancers were elevated by 30–60%, but these estimates were not statistically significant. There was more than 2-fold increased risk of multiple myeloma associated with ever use of glyphosate in adjusted analyses, although this is based on a small number of cases. The association between myeloma incidence and glyphosate exposure was consistent in both states (ever used glyphosate, fully adjusted analyses: Iowa RR = 2.6; North Carolina RR = 2.7).

Results from analyses of tertiles of increasing glyphosate exposure level are presented in Table 3. A decreased risk of lung cancer was suggested for the highest tertile of both cumulative and intensity-weighted exposure days (*p*-value for trend = 0.02); however, a similar trend was not observed in analyses using never exposed as the referent (results not shown). There was a 40% increased risk of colon cancer for the highest tertile of intensity-weighted exposure; however, no clear monotonic trend was observed for either exposure metric. Elevated risks of leukemia and pancreas cancer were observed only for the middle tertiles of both cumulative and intensity-weighted exposure days, with no increased risk among those with the highest exposure. The associations we observed in the analysis of ever use of glyphosate (Table 2) for melanoma, rectum, kidney, and bladder cancers were not confirmed in analyses based on exposure-day metrics; similarly, no exposure–response patterns were observed in analyses using never exposed as the referent or in analyses across quintiles of exposure (results not shown). No association was observed between NHL and glyphosate exposure in any analysis, including an analysis comparing the highest with the lowest quintile of exposure (> 108 vs. > 0–9 cumulative exposure days: RR = 0.9; 95% CI, 0.4–2.1).

Elevated RRs were estimated for multiple myeloma, with an approximate 2-fold increased risk for the highest tertile of both cumulative and intensity-weighted exposure days (Table 3); however, small numbers precluded precise effect estimation (*n* = 19 in adjusted analyses of exposure-day metrics). The estimated intensity-level component of the intensity-weighted exposure-day metric was not associated with multiple myeloma (highest vs. lowest tertile: RR = 0.6; 95% CI, 0.2–1.8), and observed positive associations of the intensity-weighted exposure-day metric with myeloma relied solely on the exposure-day component; therefore, only results for cumulative exposure days are shown further. When using never exposed as the referent, the association between glyphosate use and multiple myeloma was more pronounced, with more than 4-fold increased risk associated with the highest tertile of cumulative exposure days (tertile 1: RR = 2.3; 95% CI, 0.6–8.9; tertile 2: RR = 2.6; 95% CI, 0.6–11.5; tertile 3: RR = 4.4; 95% CI, 1.0–20.2; *p*-value for trend = 0.09). Although the myeloma cases were sparsely distributed in analyses of quartiles and quintiles, the highest increased risks were observed in the highest exposure categories (full set of results not shown: upper quartile vs. never exposed: RR = 6.6; 95% CI, 1.4–30.6; *p*-value for trend across quartiles = 0.01).

## Discussion

There was no association between glyphosate exposure and all cancer incidence or most of the specific cancer subtypes we evaluated, including NHL, whether the exposure metric was ever used, cumulative exposure days, or intensity-weighted cumulative exposure days. The most consistent finding in our study was a suggested association between multiple myeloma and glyphosate exposure, based on a small number of cases.

Although our study relied on self-reported exposure information, farmers have been shown to provide reliable information regarding their personal pesticide use (Blair et al. 2002; Blair and Zahm 1993; Duell et al. 2001; Engel et al. 2001; Hoppin et al. 2002). Investigators have used pesticide supplier reports (Blair and Zahm 1993) and self-reported pesticide use information provided earlier (Engel et al. 2001) to assess the validity of retrospectively reported pesticide use data. Among farmers in the AHS, Blair et al. (2002) reported high reliability for reports of ever use of a particular pesticide (ranging from 70 to > 90%). Agreement for duration and frequency of use was lower but generally 50–60% for specific pesticides. Hoppin et al. (2002) have demonstrated that farmers provide plausible data regarding lifetime duration of use, with fewer than 5% reporting implausible values for specific chemicals.

There were rather few cases of NHL for inclusion in this analysis (*n* = 92); nevertheless, the available data provided evidence of no association between glyphosate exposure and NHL incidence. This conclusion was consistent across analyses using the different exposure metrics and in analyses using either never exposed or low exposed as the referent. Furthermore, there was no apparent effect of glyphosate exposure on the risk of NHL in analyses stratified by state of residence or in analyses of highly exposed groups comparing the highest with the lowest quintile of exposure. These findings conflict with recent studies. The first report of an association of glyphosate with NHL was from a case–control study, but the estimate was based on only four exposed cases (Hardell and Eriksson 1999). A pooled analysis of this initial study with a study of hairy cell leukemia showed a relationship between glyphosate exposure and an increased risk of disease [unadjusted analysis: odds ratio (OR) = 3.0; 95% CI, 1.1–8.5] (Hardell et al. 2002). A more extensive study conducted across a large region of Canada found an elevated risk of NHL associated with glyphosate use more frequent than 2 days/year (OR = 2.1; 95% CI, 1.2–3.7) (McDuffie et al. 2001). Similarly, increased NHL risk in men was associated with having ever used glyphosate (OR = 2.1; 95% CI, 1.1–4.0) after adjustment for other commonly used pesticides in a pooled analysis of National Cancer Institute–sponsored case–control studies conducted in Nebraska, Kansas, Iowa, and Minnesota (De Roos et al. 2003b). These previous studies were retrospective in design and thereby potentially susceptible to recall bias of exposure reporting. Our analysis of the AHS cohort had a prospective design, which should largely eliminate the possibility of recall bias. Differences in recall bias could account for discrepant study results; however, evaluation of the potential for recall bias in case–control studies of pesticides among farmers has not uncovered evidence that it occurred (Blair and Zahm 1993).

Our finding of a suggested association of multiple myeloma incidence with glyphosate exposure has not been previously reported, although numerous studies have observed increased myeloma risk associated with farming occupation (Boffetta et al. 1989; Brownson et al. 1989; Cantor and Blair 1984; Cerhan et al. 1998; Cuzick and De Stavola 1988; Eriksson and Karlsson 1992; Figgs et al. 1994; Gallagher et al. 1983; La Vecchia et al. 1989; Nandakumar et al. 1986 Nandakumar et al. 1988; Pasqualetti et al. 1990; Pearce et al. 1985; Pottern et al. 1992; Reif et al. 1989; Vagero and Persson 1986). A possible biologic mechanism of how glyphosate might act along the causal pathway of this plasma cell cancer has not been hypothesized, but myeloma has been associated with agents that cause either DNA damage or immunosuppression (De Roos et al. 2003a). The association we observed was with ever use of glyphosate and cumulative exposure days of use (a combination of duration and frequency), but not with intensity of exposure. Estimated intensity of glyphosate exposure was based on general work practices that were not glyphosate specific, including the percentage of time spent mixing and applying pesticides, application method, use of personal protective equipment, and repair of pesticide application equipment (Dosemeci et al. 2002). Information on work practices specific to glyphosate use would clarify whether intensity of exposure contributes to myeloma risk.

The number of myeloma cases in our study was small, and it is plausible that spurious associations arose by chance; however, several aspects of our results argue against a chance association. The findings were internally consistent, with increased risk observed in both states. Adding to the credibility of the association, there was some indication of a dose–response relationship, with risk estimates increasing across categories of increasing exposure and stronger associations observed when using never-exposed subjects as the referent (as opposed to low exposed). Another possible explanation for spurious associations is unadjusted confounding. Our risk estimates were adjusted for some demographic and lifestyle factors and other pesticides. Of the other pesticides included in the fully adjusted model, only diazinon and trifluralin were important confounders of the glyphosate–myeloma association. It is certainly possible that an unknown risk factor for myeloma could have confounded our results; however, any unknown confounder would have to be linked with glyphosate use. Finally, the increased myeloma risk associated with glyphosate use could be due to bias resulting from a selection of subjects in adjusted analyses that differed from subjects included in unadjusted analyses. Table 1 shows that 54,315 subjects were included in age-adjusted models, whereas because of missing data for covariates, only 40,719 subjects were included in fully adjusted analyses. The association of glyphosate with myeloma differed between the two groups, even without adjustment for any covariates, with no association among the full group and a positive association among the more restricted group. Subjects who answered all the questions and were thus included in adjusted analyses differed from those who dropped out of such analyses in that they were more likely to be from Iowa (71.8% in included group vs. 44.6% in dropped group), were younger (average age, 51.5 vs. 57.9 years), and were more highly educated (46.7% educated beyond high school graduate vs. 30.2%); however, the two groups were similar in their use of glyphosate (75.9% vs. 74.5%). The increased risk associated with glyphosate in adjusted analyses may be due to selection bias or could be due to a confounder or effect modifier that is more prevalent among this restricted subgroup and is unaccounted for in our analyses. Further follow-up of the cohort and reevaluation of the association between glyphosate exposure and myeloma incidence after a greater number of cases develop will allow more detailed examination of the potential biases underlying the association.

Certain limitations of our data hinder the inferences we can make regarding glyphosate and its association with specific cancer subtypes. Although the AHS cohort is large, and there were many participants reporting glyphosate use, the small numbers of specific cancers occurring during the follow-up period hindered precise effect estimation. In addition, most applicators were male, precluding our ability to assess the association between glyphosate exposure and cancer incidence among women, for both non-sex-specific cancers and sex-specific cancers (e.g., of the breast or ovary). Our analysis provides no information on the timing of pesticide use in relation to disease, limiting the ability to sufficiently explore latency periods or effects resulting from glyphosate exposure at different ages. Despite limitations of our study, certain inferences are possible. This prospective study of cancer incidence provided evidence of no association between glyphosate exposure and most of the cancers we studied, and a suggested association between glyphosate and the risk of multiple myeloma. Future analyses within the AHS will follow up on these findings and will examine associations between glyphosate exposure and incidence of less common cancers.

## Figures and Tables

**Table 1:** Selected characteristics of applicators in the AHS by glyphosate exposure, based on data from the enrollment questionnaire (1993–1997).*^a^*

	Never exposed (*n* = 13,280)	Never exposed (*n* = 15,911)*^b^*	Higher exposed (*n* = 24,465)*^c^*
Characteristic	No. (%)	No. (%)	No. (%)
State of residence			
Iowa	9,987 (75.2)	9,785 (61.5)	15,336 (62.7)
North Carolina	3,293 (24.8)	6,126 (38.5)	9,129 (37.3)
Age (years)			
< 40	2,279 (17.2)	2,226 (14.0)	4,190 (17.1)
40–49	3,420 (25.8)	4,279 (26.9)	7,899 (32.3)
50–59	2,989 (22.5)	3,931 (24.7)	6,035 (24.7)
60–69	2,715 (20.4)	3,266 (20.5)	3,997 (16.3)
70	1,877 (14.1)	2,209 (13.9)	2,344 (9.6)
Sex			
Male	12,778 (96.2)	15,505 (97.5)	23,924 (97.8)
Female	502 (3.8)	406 (2.6)	541 (2.2)
Applicator type*^d^*			
Private	12,067 (90.9)	15,008 (94.3)	21,938 (89.7)
Commercial	1,213 (9.1)	903 (5.7)	2,527 (10.3)
Education			
High school graduate or GED	8,898 (68.7)	8,997 (57.9)	11,975 (50.1)
Beyond high school	4,060 (31.3)	6,530 (42.1)	11,936 (49.9)
Smoking history			
Never	7,298 (57.3)	8,241 (53.2)	12,751 (53.7)
≤ 12 pack-years	2,866 (22.5)	3,597 (23.2)	5,572 (23.5)
> 12 pack-years	2,567 (20.2)	3,643 (23.5)	5,439 (22.9)
Alcohol consumption in past year			
None	4,087 (32.7)	5,352 (35.6)	7,023 (29.8)
≤ 6 drinks/month	4,461 (35.7)	5,291 (35.2)	8,149 (34.5)
> 6 drinks/month	3,936 (31.5)	4,387 (29.2)	8,422 (35.7)
Family history of cancer			
No	8,701 (65.5)	9,520 (59.8)	14,668 (60.0)
Yes	4,579 (34.5)	6,391 (40.2)	9,797 (40.0)
Use of other common pesticides			
2,4-D	7,030 (53.3)	11,879 (75.2)	20,699 (85.1)
Alachlor	4,896 (39.7)	7,321 (50.9)	13,790 (59.7)
Atrazine	7,707 (58.5)	10,533 (66.6)	18,237 (75.0)
Metolachlor	3,890 (31.6)	6,172 (43.1)	12,952 (56.2)
Trifluralin	4,239 (34.0)	7,109 (49.7)	14,675 (63.5)
Carbaryl	4,110 (33.7)	8,515 (58.1)	15,139 (64.8)
Benomyl	510 (4.3)	1,418 (9.9)	3,391 (14.8)
Maneb	492 (4.1)	1,412 (9.9)	2,929 (12.9)
Paraquat	1,067 (9.0)	3,021 (21.2)	8,031 (35.2)
Diazinon	1,906 (16.0)	4,615 (32.4)	9,107 (40.0)

a Includes observations for subjects included in age-adjusted Poisson regression models of cancer incidence (*n* = 54,315).

b Lowest tertile of cumulative exposure days.

c Highest two tertiles of cumulative exposure days; the sum of the three tertiles of cumulative exposure days (*n* = 40,376) does not equal the total number of subjects who reported having ever used glyphosate (*n* = 41,035) because of missing data on duration and frequency of use.

d “Private” refers primarily to individual farmers, and “commercial” refers to professional pesticide applicators.

**Table 2:** Association of glyphosate exposure (ever/never used) with common cancers*^a^* among AHS applicators.

			RR (95% CI)*^b^*	
Cancer site	Total no. of cancers*^c^*	Ever used glyphosate (% of total)	Effect estimates adjusted for age (*n* = 54,315)*^d^*	Adjusted for age, demographic and lifestyle factors,and other pesticides*^d^*
All cancers	2,088	73.6	1.0 (0.9–1.1)	1.0 (0.9–1.2)
Lung	204	72.1	1.0 (0.7–1.3)	0.9 (0.6–1.3)
Oral cavity	59	76.3	1.1 (0.6–2.0)	1.0 (0.5–1.8)
Colon	174	75.3	1.1 (0.8–1.6)	1.4 (0.8–2.2)*^e^*
Rectum	76	77.6	1.2 (0.7–2.1)	1.3 (0.7–2.3)
Pancreas	38	76.3	1.2 (0.6–2.5)	0.7 (0.3–2.0)*^e^*
Kidney	63	73.0	1.0 (0.6–1.7)	1.6 (0.7–3.8)*^e^*
Bladder	79	76.0	1.2 (0.7–2.0)	1.5 (0.7–3.2)*^e^*
Prostate	825	72.5	1.0 (0.8–1.1)	1.1 (0.9–1.3)
Melanoma	75	84.0	1.8 (1.0–3.4)	1.6 (0.8–3.0)
All lymphohematopoietic cancers	190	75.3	1.1 (0.8–1.5)	1.1 (0.8–1.6)
NHL	92	77.2	1.2 (0.7–1.9)	1.1 (0.7–1.9)
Leukemia	57	75.4	1.1 (0.6–2.0)	1.0 (0.5–1.9)
Multiple myeloma	32	75.0	1.1 (0.5–2.4)	2.6 (0.7–9.4)*^f^*

a Cancers for which at least 30 subjects had sufficient information for inclusion in age-adjusted analyses.

b RRs and 95% CIs from Poisson regression models.

c Frequencies among subjects included in age-adjusted analyses.

d Numbers of subjects in these analyses are lower than in age-adjusted analyses because of missing observations for some covariates (models adjusted for demographic and lifestyle factors include 49,211 subjects; models additionally adjusted for other pesticides include 40,719 subjects).

e Estimates adjusted for other pesticides are shown because inclusion of other pesticide variables in the model changed the effect estimate for glyphosate by at least 20%.

f The estimate for myeloma was not confounded by other pesticides according to our change-in-estimate rule of ≥20%; however, the fully adjusted estimate is shown for the purpose of comparison with state-specific estimates (in the text), which were confounded by other pesticides and required adjustment.

**Table 3:** Association of glyphosate exposure (cumulative exposure days and intensity-weighted exposure days) with common cancers*^a^* among AHS applicators.

	Cumulative exposure days*^b^*				Intensity-weighted exposure days*^c^*			
Cancer site	Tertile cut points	No.	RR (95% CI)*^d^*	*p*-Trend	Tertile cut points	No.	RR (95% CI)*^d^*	*p*-Trend
All cancers	1–20	594	1.0		0.1–79.5	435	1.0	
	21–56	372	1.0 (0.9–1.1)		79.6–337.1	436	0.9 (0.8–1.0)	
	57–2,678	358	1.0 (0.9–1.1)	0.57	337.2–18,241	438	0.9 (0.8–1.1)	0.35
Lung	1–20	40	1.0		0.1–79.5	27	1.0	
	21–56	26	0.9 (0.5–1.5)*^e^*		79.6–337.1	38	1.1 (0.7–1.9)*^e^*	
	57–2,678	26	0.7 (0.4–1.2)*^e^*	0.21	337.2–18,241	27	0.6 (0.3–1.0)*^e^*	0.02
Oral cavity	1–20	18	1.0		0.1–79.5	11	1.0	
	21–56	10	0.8 (0.4–1.7)		79.6–337.1	14	1.1 (0.5–2.5)	
	57–2,678	10	0.8 (0.4–1.7)	0.66	337.2–18,241	13	1.0 (0.5–2.3)	0.95
Colon	1–20	32	1.0		0.1–79.5	25	1.0	
	21–56	28	1.4 (0.9–2.4)*^e^*		79.6–337.1	20	0.8 (0.5–1.5)*^c^*	
	57–2,678	15	0.9 (0.4–1.7)*^e^*	0.54	337.2–18,241	30	1.4 (0.8–2.5)*^c^*	0.10
Rectum	1–20	20	1.0		0.1–79.5	16	1.0	
	21–56	17	1.3 (0.7–2.5)		79.6–337.1	18	1.0 (0.5–2.0)	
	57–2,678	14	1.1 (0.6–2.3)	0.70	337.2–18,241	16	0.9 (0.5–1.9)	0.82
Pancreas	0–20	9	1.0		0–79.5	6	1.0	
	21–56	9	1.6 (0.6–4.1)		79.6–337.1	16	2.5 (1.0–6.3)	
	57–2,678	7	1.3 (0.5–3.6)	0.83	337.2–18,241	3	0.5 (0.1–1.9)	0.06
Kidney	1–20	20	1.0		0.1–79.5	20	1.0	
	21–56	8	0.6 (0.3–1.4)		79.6–337.1	7	0.3 (0.1–0.7)	
	57–2,678	9	0.7 (0.3–1.6)	0.34	337.2–18,241	10	0.5 (0.2–1.0)	0.15
Bladder	1–20	23	1.0		0.1–79.5	14	1.0	
	21–56	14	1.0 (0.5–1.9)		79.6–337.1	8	0.5 (0.2–1.3)	
	57–2,678	17	1.2 (0.6–2.2)	0.53	337.2–18,241	13	0.8 (0.3–1.8)	0.88
Prostate	1–20	239	1.0		0.1–79.5	167	1.0	
	21–56	132	0.9 (0.7–1.1)		79.6–337.1	169	1.0 (0.8–1.2)	
	57–2,678	145	1.1 (0.9–1.3)	0.69	337.2–18,241	174	1.1 (0.9–1.3)	0.60
Melanoma	1–20	23	1.0		0.1–79.5	24	1.0	
	21–56	20	1.2 (0.7–2.3)		79.6–337.1	16	0.6 (0.3–1.1)	
	57–2,678	14	0.9 (0.5–1.8)	0.77	337.2–18,241	17	0.7 (0.3–1.2)	0.44
All lymphohematopoietic cancers	1–20	48	1.0		0.1–79.5	38	1.0	
	21–56	38	1.2 (0.8–1.8)		79.6–337.1	40	1.0 (0.6–1.5)	
	57–2,678	36	1.2 (0.8–1.8)	0.69	337.2–18,241	43	1.0 (0.7–1.6)	0.90
NHL	1–20	29	1.0		0.1–79.5	24	1.0	
	21–56	15	0.7 (0.4–1.4)		79.6–337.1	15	0.6 (0.3–1.1)	
	57–2,678	17	0.9 (0.5–1.6)	0.73	337.2–18,241	22	0.8 (0.5–1.4)	0.99
Leukemia	1–20	9	1.0		0.1–79.5	7	1.0	
	21–56	14	1.9 (0.8–4.5)*^e^*		79.6–337.1	17	1.9 (0.8–4.7)*^e^*	
	57–2,678	9	1.0 (0.4–2.9)*^e^*	0.61	337.2–18,241	8	0.7 (0.2–2.1)*^e^*	0.11
Multiple myeloma	1–20	8	1.0		0–79.5	5	1.0	
	21–56	5	1.1 (0.4–3.5)*^e^*		79.6–337.1	6	1.2 (0.4–3.8)*^e^*	
	57–2,678	6	1.9 (0.6–6.3)*^e^*	0.27	337.2–18,241	8	2.1 (0.6–7.0)*^e^*	0.17

a Cancers for which at least 30 subjects had sufficient information for inclusion in age-adjusted analyses.

b Numbers of subjects in analyses vary depending on missing observations for cumulative exposure days and some covariates (models adjusted for demographic and lifestyle factors include 36,823 subjects; models additionally adjusted for other pesticides include 30,699 subjects).

c Numbers of subjects in analyses vary depending on missing observations for intensity-weighted cumulative exposure days and some covariates (models adjusted for demographic and lifestyle factors include 36,509 subjects; models additionally adjusted for other pesticides include 30,613 subjects).

d Relative rate ratios and 95% CIs from Poisson regression analyses.

e Estimates adjusted for other pesticides are shown because inclusion of other pesticide variables in the model changed the effect estimate for glyphosate by at least 20%.

## References

[b1-ehp0113-000049] Alavanja MC, Sandler DP, McMaster SB, Zahm SH, McDonnell CJ, Lynch CF (1996). The Agricultural Health Study. Environ Health Perspect.

[b2-ehp0113-000049] Blair A, Tarone R, Sandler D, Lynch CF, Rowland A, Wintersteen W (2002). Reliability of reporting on lifestyle and agricultural factors by a sample of participants in the Agricultural Health Study from Iowa. Epidemiology.

[b3-ehp0113-000049] Blair A, Zahm SH (1993). Patterns of pesticide use among farmers: implications for epidemiologic research. Epidemiology.

[b4-ehp0113-000049] Boffetta P, Stellman SD, Garfinkel L (1989). A case-control study of multiple myeloma nested in the American Cancer Society prospective study. Int J Cancer.

[b5-ehp0113-000049] Boffetta P, Bolognesi C, Bonatti S, Degan P, Gallerani E, Peluso M, Rabboni R Genotoxic activity of glyphosate and its technical formulation Roundup. J Agric Food Chem.

[b6-ehp0113-000049] Brownson RC, Reif JS, Chang JC, Davis JR (1989). Cancer risks among Missouri farmers. Cancer.

[b7-ehp0113-000049] Cantor KP, Blair A (1984). Farming and mortality from multiple myeloma: a case-control study with the use of death certificates. J Natl Cancer Inst.

[b8-ehp0113-000049] Cerhan JR, Cantor KP, Williamson K, Lynch CF, Torner JC, Burmeister LF (1998). Cancer mortality among Iowa farmers: recent results, time trends, and lifestyle factors (United States). Cancer Causes Control.

[b9-ehp0113-000049] Cuzick J, De Stavola B (1988). Multiple myeloma—a case-control study. Br J Cancer.

[b10-ehp0113-000049] Daruich J, Zirulnik F, Gimenez MS (2001). Effect of the herbicide glyphosate on enzymatic activity in pregnant rats and their fetuses. Environ Res.

[b11-ehp0113-000049] De Roos AJ Baris D Weiss NS Herrinton LJ 2003a. Epidemiology of multiple myeloma. In: Myeloma: Biology and Management (Malpas JS, Bergsagel DE, Kyle RA, Anderson KC, eds). 3rd ed. Philadelphia:Saunders, 117–158.

[b12-ehp0113-000049] De Roos AJ Zahm SH Cantor KP Weisenburger DD Holmes FF Burmeister LF 2003b. Integrative assessment of multiple pesticides as risk factors for non-Hodgkin’s lymphoma among men. Occup Environ Med 60:E11. Available: http://oem.bmjjournals.com/cgi/content/full/60/9/e11 [accessed 30 November 2004]. 10.1136/oem.60.9.e11 PMC1740618 12937207

[b13-ehp0113-000049] Dosemeci M, Alavanja MC, Rowland AS, Mage D, Zahm SH, Rothman N (2002). A quantitative approach for estimating exposure to pesticides in the Agricultural Health Study. Ann Occup Hyg.

[b14-ehp0113-000049] Duell EJ, Millikan RC, Savitz DA, Schell MJ, Newman B, Tse CJ (2001). Reproducibility of reported farming activities and pesticide use among breast cancer cases and controls. A comparison of two modes of data collection. Ann Epidemiol.

[b15-ehp0113-000049] El Demerdash FM, Yousef MI, Elagamy EI (2001). Influence of paraquat, glyphosate, and cadmium on the activity of some serum enzymes and protein electrophoretic behavior (in vitro). J Environ Sci Health B.

[b16-ehp0113-000049] Engel LS, Seixas NS, Keifer MC, Longstreth WT, Checkoway H (2001). Validity study of self-reported pesticide exposure among orchardists. J Expo Anal Environ Epidemiol.

[b17-ehp0113-000049] Eriksson M, Karlsson M (1992). Occupational and other environmental factors and multiple myeloma: a population based case-control study. Br J Ind Med.

[b18-ehp0113-000049] Figgs LW, Dosemeci M, Blair A (1994). Risk of multiple myeloma by occupation and industry among men and women: a 24-state death certificate study. J Occup Med.

[b19-ehp0113-000049] Folmar LC, Sanders HO, Julin AM (1979). Toxicity of the herbicide glyphosphate and several of its formulations to fish and aquatic invertebrates. Arch Environ Contam Toxicol.

[b20-ehp0113-000049] Gallagher RP, Spinelli JJ, Elwood JM, Skippen DH (1983). Allergies and agricultural exposure as risk factors for multiple myeloma. Br J Cancer.

[b21-ehp0113-000049] Garry VF, Burroughs B, Tarone R, Kesner JS (1999). Herbicides and adjuvants: an evolving view. Toxicol Ind Health.

[b22-ehp0113-000049] Grisolia CK (2002). A comparison between mouse and fish micronucleus test using cyclophosphamide, mitomycin C and various pesticides. Mutat Res.

[b23-ehp0113-000049] Hardell L, Eriksson M (1999). A case-control study of non-Hodgkin lymphoma and exposure to pesticides. Cancer.

[b24-ehp0113-000049] Hardell L, Eriksson M, Nordstrom M (2002). Exposure to pesticides as risk factor for non-Hodgkin’s lymphoma and hairy cell leukemia: pooled analysis of two Swedish case-control studies. Leuk Lymphoma.

[b25-ehp0113-000049] Hietanen E, Linnainmaa K, Vainio H (1983). Effects of phenoxy-herbicides and glyphosate on the hepatic and intestinal biotransformation activities in the rat. Acta Pharmacol Toxicol (Copenh).

[b26-ehp0113-000049] Hoppin JA, Yucel F, Dosemeci M, Sandler DP (2002). Accuracy of self-reported pesticide use duration information from licensed pesticide applicators in the Agricultural Health Study. J Expo Anal Environ Epidemiol.

[b27-ehp0113-000049] Kale PG, Petty BT, Walker S, Ford JB, Dehkordi N, Tarasia S (1995). Mutagenicity testing of nine herbicides and pesticides currently used in agriculture. Environ Mol Mutagen.

[b28-ehp0113-000049] La Vecchia C, Negri E, D’Avanzo B, Franceschi S (1989). Occupation and lymphoid neoplasms. Br J Cancer.

[b29-ehp0113-000049] Li AP, Long TJ (1988). An evaluation of the genotoxic potential of glyphosate. Fundam Appl Toxicol.

[b30-ehp0113-000049] Lioi MB, Scarfi MR, Santoro A, Barbieri R, Zeni O, Di Berardino D (1998a). Genotoxicity and oxidative stress induced by pesticide exposure in bovine lymphocyte cultures in vitro. Mutat Res.

[b31-ehp0113-000049] Lioi MB, Scarfi MR, Santoro A, Barbieri R, Zeni O, Salvemini F (1998b). Cytogenetic damage and induction of pro-oxidant state in human lymphocytes exposed in vitro to gliphosate, vinclozolin, atrazine, and DPX-E9636. Environ Mol Mutagen.

[b32-ehp0113-000049] Martinez TT, Long WC, Hiller R (1990). Comparison of the toxicology of the herbicide Roundup by oral and pulmonary routes of exposure. Proc West Pharmacol Soc.

[b33-ehp0113-000049] McDuffie HH, Pahwa P, McLaughlin JR, Spinelli JJ, Fincham S, Dosman JA (2001). Non-Hodgkin’s lymphoma and specific pesticide exposures in men: cross-Canada study of pesticides and health. Cancer Epidemiol Biomarkers Prev.

[b34-ehp0113-000049] Mitchell DG, Chapman PM, Long TJ (1987). Acute toxicity of Roundup and Rodeo herbicides to rainbow trout, chinook, and coho salmon. Bull Environ Contam Toxicol.

[b35-ehp0113-000049] Moriya M, Ohta T, Watanabe K, Miyazawa T, Kato K, Shirasu Y (1983). Further mutagenicity studies on pesticides in bacterial reversion assay systems. Mutat Res.

[b36-ehp0113-000049] Nandakumar A, Armstrong BK, de Klerk NH (1986). Multiple myeloma in Western Australia: a case-control study in relation to occupation, father’s occupation, socioeconomic status and country of birth. Int J Cancer.

[b37-ehp0113-000049] Nandakumar A, English DR, Dougan LE, Armstrong BK (1988). Incidence and outcome of multiple myeloma in Western Australia, 1960 to 1984. Aust NZ J Med.

[b38-ehp0113-000049] National Center for Health Statistics 1999. National Death Index Homepage. Hyattsville, MD:National Center for Health Statistics. Available: http://www.cdc.gov/nchs/r&d/ndi/ndi.htm [accessed 30 November 2004].

[b39-ehp0113-000049] National Institutes of Health 2004. Agricultural Health Study Homepage. Bethesda, MD:National Institutes of Health. Available: http://www.aghealth.org [accessed 25 September 2004].

[b40-ehp0113-000049] Olorunsogo OO, Bababunmi EA, Bassir O (1979). Effect of glyphosate on rat liver mitochondria in vivo. Bull Environ Contam Toxicol.

[b41-ehp0113-000049] Pasqualetti P, Casale R, Collacciani A, Colantonio D (1990). Work activities and the risk of multiple myeloma. A case-control study. Med Lav.

[b42-ehp0113-000049] Pearce NE, Smith AH, Fisher DO (1985). Malignant lymphoma and multiple myeloma linked with agricultural occupations in a New Zealand Cancer Registry-based study. Am J Epidemiol.

[b43-ehp0113-000049] Peluso M, Munnia A, Bolognesi C, Parodi S (1998). ^32^P-Postlabeling detection of DNA adducts in mice treated with the herbicide Roundup. Environ Mol Mutagen.

[b44-ehp0113-000049] Pottern LM, Heineman EF, Olsen JH, Raffn E, Blair A (1992). Multiple myeloma among Danish women: employment history and workplace exposures. Cancer Causes Control.

[b45-ehp0113-000049] Rank J, Jensen AG, Skov B, Pedersen LH, Jensen K (1993). Genotoxicity testing of the herbicide Roundup and its active ingredient glyphosate isopropylamine using the mouse bone marrow micronucleus test, Salmonella mutagenicity test, and Allium anaphase-telophase test. Mutat Res.

[b46-ehp0113-000049] Reif J, Pearce N, Fraser J (1989). Cancer risks in New Zealand farmers. Int J Epidemiol.

[b47-ehp0113-000049] Steinrucken HC, Amrhein N (1980). The herbicide glyphosate is a potent inhibitor of 5-enolpyruvyl-shikimic acid-3-phosphate synthase. Biochem Biophys Res Commun.

[b48-ehp0113-000049] U.S. EPA 1993. U.S. Environmental Protection Agency Reregistration Eligibility Decision (RED) Glyphosate. EPA-738-R-93-014. Washington, DC:U.S. Environmental Protection Agency.

[b49-ehp0113-000049] Vagero D, Persson G (1986). Occurrence of cancer in socioeconomic groups in Sweden. An analysis based on the Swedish Cancer Environment Registry. Scand J Soc Med.

[b50-ehp0113-000049] Vigfusson NV, Vyse ER (1980). The effect of the pesticides, Dexon, Captan and Roundup, on sister-chromatid exchanges in human lymphocytes in vitro. Mutat Res.

[b51-ehp0113-000049] Walsh LP, McCormick C, Martin C, Stocco DM (2000). Roundup inhibits steroidogenesis by disrupting steroidogenic acute regulatory (StAR) protein expression. Environ Health Perspect.

[b52-ehp0113-000049] WHO 1977. International Classification of Diseases: Manual of the International Statistical Classification of Diseases, Injuries, and Causes of Death, Vol 1, 9th revision. Geneva:World Health Organization.

[b53-ehp0113-000049] WHO 1994. International Programme on Chemical Safety. Glyphosate. Environmental Health Criteria 159. Geneva:World Health Organization.

[b54-ehp0113-000049] Wildeman AG, Nazar RN (1982). Significance of plant metabolism in the mutagenicity and toxicity of pesticides. Can J Genet Cytol.

[b55-ehp0113-000049] Williams GM, Kroes R, Munro IC (2000). Safety evaluation and risk assessment of the herbicide Roundup and its active ingredient, glyphosate, for humans. Regul Toxicol Pharmacol.

[b56-ehp0113-000049] Yousef MI, Salem MH, Ibrahim HZ, Helmi S, Seehy MA, Bertheussen K (1995). Toxic effects of carbofuran and glyphosate on semen characteristics in rabbits. J Environ Sci Health B.

